# Effects of Creatine Treatment on Jejunal Phenotypes in a Rat Model of Acidosis

**DOI:** 10.3390/antiox8070225

**Published:** 2019-07-17

**Authors:** Chiara Sironi, Francesca Bodega, Luciano Zocchi, Cristina Porta

**Affiliations:** Dipartimento di Fisiopatologia Medico-Chirurgica e dei Trapianti, Facoltà di Medicina e Chirurgia, Università degli Studi di Milano, Via Mangiagalli 32, 20133 Milano, Italy

**Keywords:** acidosis, antioxidant enzymes, creatine, heat shock proteins, malondialdehyde, oxidative stress

## Abstract

We investigated the effects of creatine treatment on jejunal phenotypes in a rat model of oxidative stress induced by acidosis. In particular, the activities of some antioxidant enzymes (superoxide dismutase, glutathione peroxidase, catalase, and glutathione reductase), the level of lipid peroxidation, the expression of heat shock proteins (HSP70), and the expression of the major carriers of the cells (Na^+^/K^+^-ATPase, sodium-glucose Transporter 1—SGLT1, and glucose transporter 2—GLUT2) were measured under control and chronic acidosis conditions. Creatine did not affect the activity of antioxidant enzymes in either the control or acidosis groups, except for catalase, for which the activity was reduced in both conditions. Creatine did not change the lipid peroxidation level or HSP70 expression. Finally, creatine stimulated (Na^+^/K^+^)-ATPase expression under both control and chronic acidosis conditions. Chronic acidosis caused reductions in the expression levels of GLUT2 and SGLT1. GLUT2 reduction was abolished by creatine, while the presence of creatine did not induce any strengthening effect on the expression of SGLT1 in either the control or chronic acidosis groups. These results indicate that creatine has antioxidant properties that are realized through direct interaction of the molecule with reactive oxygen species. Moreover, the administration of creatine seems to determine a functional strengthening of the tissue, making it more resistant to acidosis.

## 1. Introduction

Creatine (Cr, N-[aminoiminomethyl]-N-methyl glycine) is an endogenous amino acid produced in the liver, kidneys, and pancreas starting from glycine, methionine, and arginine [1]. In mammals, creatine is also obtained from the diet in meat-containing products [2,3]. Under normal conditions, dietary intake supplies about 50% of creatine requirements [2,3]. Creatine is then transported via the blood to the tissues and absorbed into the cells against its concentration gradient by a specific transporter [4,5,6]. As well as being an ergogenic aid that improves exercise performance in athletes, creatine is increasingly being used as a possible dietary supplement for the treatment of various diseases such as myopathies, neurodegenerative disorders, cancer, rheumatic diseases, and type 2 diabetes [7,8,9,10,11]. The neuroprotective potential of creatine has been illustrated in numerous models of neurodegeneration as well as in animal and human models of traumatic brain injury and cerebral oxygen deprivation [12,13,14,15]. Moreover, creatine has been shown to maintain intestinal homeostasis and protect against colitis [16]. For instance, in mouse colitis models, creatine supplementation has been shown to attenuate the inflammatory response [17]. The beneficial effect of creatine seems to be due to its ability of buffering cellular ATP levels, which reduction leads to the formation of reactive oxygen species (ROS), with consequent oxidative damage [3]. Indeed, oxidative stress has been stably recognized as one of the multiple etiological factors involved in these pathologies. In fact, in vitro studies have revealed that creatine may have direct antioxidant properties by acting as a scavenger of free radicals [18,19]. In addition, studies show that creatine affects oxidative stress parameters, such as lipid peroxidation, in the livers of mice treated with pravastatin [20].

Acidosis promotes lipid peroxidation or other manifestations of oxidant-mediated damage in various cell types [21,22,23,24]. Moreover, a number of studies indicate that acidosis is involved in ROS-induced intestinal inflammatory diseases [25,26,27]. In turn, the acidosis associated with inflammatory conditions produces oxidative stress and/or amplifies its effects [6,23,28,29,30]. In vivo and in vitro studies indicate that at an acidotic pH, the response of the gut to an insult is magnified [31].

The gastrointestinal mucosa is repetitively exposed to oxidative stress exerted by luminal oxidants ingested with food, despite its mucus lining shows antioxidant properties. Much evidence has suggested that oxidant agents not only determine cytotoxicity, but they also play an important role in mediating specific cell responses and gene expressions involved in degenerative pathophysiologic conditions, such as inflammation and cancer. Reactive oxygen species are implicated in the pathogenesis of various gastrointestinal diseases, including post-ischemic reperfusion injury of the small intestine, gluten-related disorders [32], gastric ulcers [26,33], ulcerative colitis [34], Crohn’s disease [35], and cancer and inflammation [36,37]. Antioxidants play a crucial role in preventing damage induced by oxidative stress through the neutralization of free radicals. The aim of this study is, therefore, to show if creatine supplementation in vivo should ameliorate the antioxidant response of intestinal cells and prevent intestinal tissue injury induced by oxidative stress.

Previous experiments have shown that a creatine transporter operates at the brush border level of the rat jejunal enterocyte [4,38]. Moreover, in vitro treatment with creatine had positive effects on rat jejunal epithelium under conditions of oxidative stress induced by an ischemia and reperfusion model in vitro [39]. In the present study, the effect of creatine was studied in vivo, by a subministration of creatine lasting for 11 days, while oxidative stress was induced by acidosis. We measured various oxidant and antioxidant parameters on cells extracted from rat jejunums after administration of creatine under control and chronic acidosis conditions. In particular, we investigated whether creatine administration had effects on the activities of the main antioxidant enzymes of the cell and whether creatine could affect parameters associated with oxidative stress, such as the level of lipid peroxidation. Furthermore, to investigate the effects of the molecule on intestinal function, the expression levels of (Na^+^/K^+^)-ATPase, sodium-glucose transporter 1 (SGLT1), and glucose transporter 2 (GLUT2) were examined. Finally, to assess whether the presence of creatine may have any cytoprotective effects in relation to stress conditions, we measured the expression of heat shock proteins (HSP70), which are known to play a protective role against thermal and oxidative stress in intestinal epithelial cells.

## 2. Materials and Methods

The experiments were performed according to national ethical guidelines and were approved by “Comune di Milano—Uff. Diritti degli animali”, “Regione Lombardia” and “Ministero della Salute” (prot. 5/2008). Male albino rats (Wistar strain, Charles River Italiana) weighing 250–300 g (about two months old) were used.

Experiments were performed on 16 rats that were maintained on standard chow with access to drinking water ad libitum. To induce metabolic acidosis, rats were given 0.28 M NH_4_Cl in drinking water for 7 d. Four different experimental conditions were set up: (1) control, (2) creatine, (3) NH_4_Cl, and (4) creatine + NH_4_Cl. For each condition, rats were watered with 75 mL of the respective solutions, which were all prepared using tap water. Net water was administered to the first group, to the second a 20 mM creatine monohydrate solution, to the third a 280 mM NH_4_Cl solution, while to the fourth a solution of NH_4_Cl 280 mM and 20 mM creatine was added. For the first two conditions, the animals were treated for a total of 11 d. For the last two conditions, the above-described treatment lasted a total of 11 d and was preceded by a four-day pretreatment protocol in which rats were given only water (third condition) or a 20 mM solution of creatine monohydrate (fourth condition). The body weights of the animals were recorded on the first and last days. After treatment, animals were killed under anesthesia, always between 9:00 and 10:00 a.m., to avoid any possible cyclic daily variations in antioxidant levels. To confirm acidosis, blood pH was measured immediately before death directly from blood in the left ventricle. The intestinal tissues were dissected, the jejunums were resected, and the mucosa was scraped, weighed, rapidly freeze-clamped at liquid nitrogen temperature, and stored at −80 °C until use.

### 2.1. Enzyme Activities

The jejunal scraped material was homogenized in 50 mM potassium phosphate buffer (pH 7.4) containing 1 mM ethylenediamine tetra-acetic acid (EDTA). The samples were centrifuged for 10 min at 12,000× *g*, 4 °C, and the supernatant was used for activity assays of enzymes. All enzyme activities are expressed as mU/mg proteins.

Catalase (CAT) activity was measured according to the method of Aebi [40] by measuring the decrease in absorbance of H_2_O_2_ at 240 nm for 5 min.

Superoxide dismutase (SOD) activity was measured by the inhibition of pyrogallol autoxidation at 420 nm according to Guzik et al. [41].

Glutathione peroxidase (GPx) activity was measured by following the oxidation of nicotinamide adenine dinucleotide phosphate (NADPH) at 340 nm according to Anwer et al. [42].

Glutathione reductase (GR) activity was measured as a decrease in the absorbance of NADPH for 5 min at 340 nm according to Ojano-Dirain et al. [43].

### 2.2. Lipid Peroxidation

The jejunal scraped material was homogenized in 1.15% KCl and centrifuged for 10 min at 12000× *g* and 4 °C. Malondialdehyde (MDA) production, expressed as mU/mg protein, was assessed spectrophotometrically on the supernatant with the method defined by Ohkawa et al. [44].

### 2.3. Protein Extraction and Western Blot

The jejunal scraped material from each rat was resuspended in cold buffer sucrose-histidine (IS) containing 0.3 M sucrose, 25 mM histidine, and 1 mM EDTA, supplemented with protease inhibitors (Roche, Monza, Italy). This was homogenized and then centrifuged at 4 °C for 15 min at 5000× *g*. The supernatant was recovered; protein concentration was measured according to the Bradford method [45], and equal amounts of protein (5 µg for Na^+^/K^+^-ATPase and 60 µg for GLUT2, SGLT1, and HSP70) were analyzed on the same SDS-PAGE (sodium dodecyl sulfate–polyacrylamide gel electrophoresis). Each sample was dissolved in Laemmli sample buffer (final concentration 2% (*w*/*v*) sodium dodecyl sulfate (SDS), 50% (*v*/*v*) glycerol, 1% (*v*/*v*) 2-mercaptoethanol, 50 mM Tris, pH 6.8) and heated at 65 °C for 10 min. A 7% polyacrylamide mini-gel was run in a mini-gel apparatus (Miniprotean 3, Biorad, Segrate, Italy) for 2 h at 120 V. Proteins were electrophoretically transferred to a polyvinylidene difluoride (PVDF) membrane. After blocking with 5% nonfat dry milk in Tris-buffered saline with Tween (TBST) buffer (50 mM Tris, 150 mM NaCl, 0.1% Tween, pH 8) for 2 h at room temperature, the proteins were probed overnight at 4 °C with the specific primary antibodies. Specifically, the antibodies used were: anti-ATPase alpha 1 (Na^+^/K^+^) (Novus Biologicals, Centennial, CO, USA) diluted 1:5000, polyclonal anti-GLUT2 (Chemicon, Temecula, CA, USA) diluted 1:1000, anti-SGLT1 (Millipore, Burlington, MA, USA) diluted 1:1000, and anti HSPA1A-Heat shock protein 70 kDa protein 1A (Aviva, San Diego, CA, USA) diluted 1:5000. The primary antibodies for GLUT2, SGLT1, and HSP70 were detected with goat anti-rabbit IgG conjugated to horseradish peroxidase (Chemicon, Temecula, CA, USA) diluted 1:40,000. Anti-ATPase alpha 1 (Na^+^, K^+^) was detected with goat anti-mouse IgG conjugated to horseradish peroxidase (Santa Cruz Biotech, Dallas, TX, USA) diluted 1:3000. All primary and secondary antibodies were diluted in 5% nonfat dry milk TBST buffer (Tris-buffered saline with Tween). Sites of antibody–antigen reactions were visualized using Amersham ECL Plus (Amersham, Cologno Monzese, Italy), according to the manufacturer’s instructions, before exposure to X-ray film (Celbio, Pero, Italy). After autoradiography, the ratio among different experimental conditions was determined on each CL-Xposure film. The densitometric analysis was conducted in a blinded fashion by two researchers who independently chose the regions of interest by analyzing western blot signals. The chosen areas were numerically integrated and measured with ImageJ Tool software (version 1.52d, National Institutes of Health, Bethesda, MD, USA). The data shown are the means of 4 different experiments. To assess equal loading of the lanes, the quality of the electrophoretic run, and the efficiency of the transfer, the electrophoresis gel and the blotted membrane were stained with 0.25% Coomassie Blue and 0.1% Ponceau in acetic acid, respectively [46].

### 2.4. Statistics

Statistical analysis was performed by Student’s *t* test or by analysis of variance (ANOVA) followed by post hoc Tukey’s limitation. Values are reported as means ± S.E.

## 3. Results and Discussion

### 3.1. Blood pH and Body Weight

**Figure 1:**
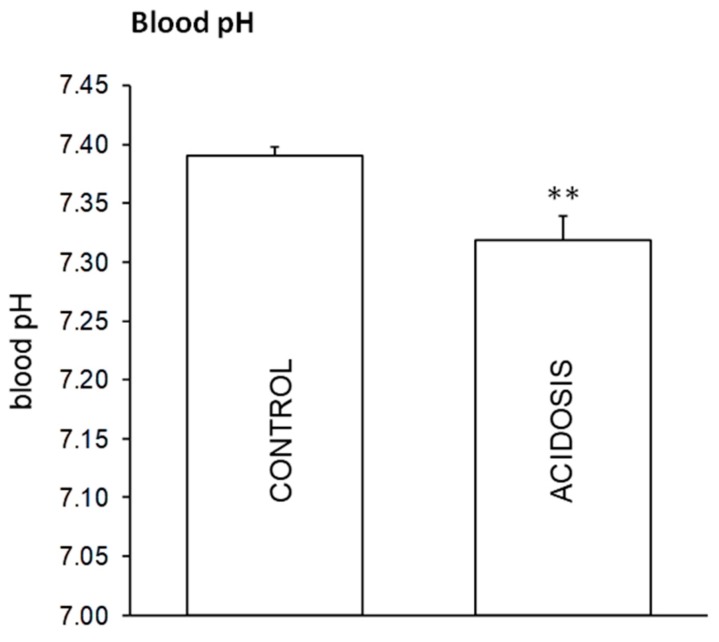
Blood pH values after the induction of metabolic acidosis by administration of 0.28 M NH_4_Cl in drinking water for 7 d. Values are means ± S.E. ** *p* ≤ 0.05 vs control. Number of experiments = 16.

**Figure 2:**
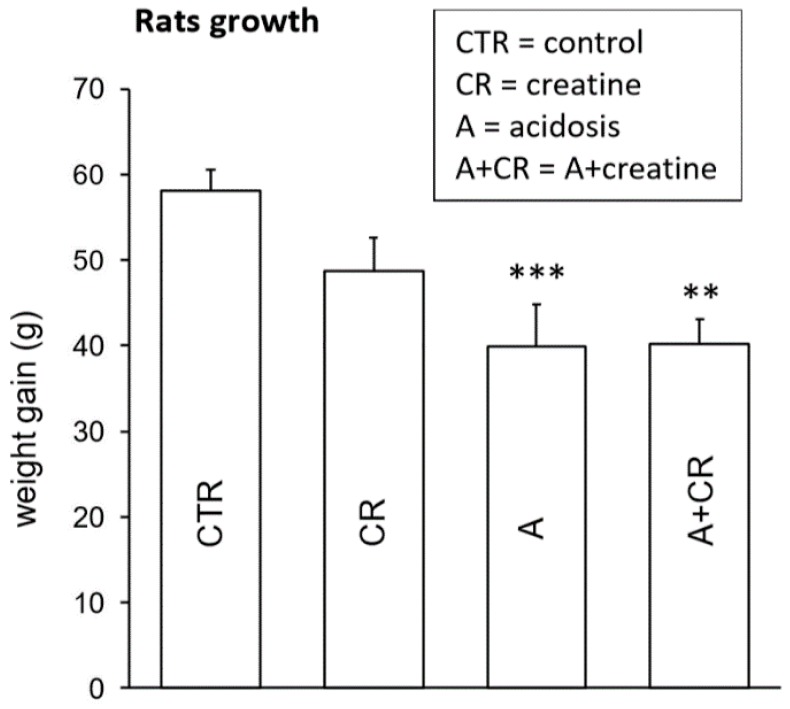
Rat weight (g) gain after 11 d of treatment. Values are means ± S.E. *** *p* ≤ 0.05 vs. control. ** *p* ≤ 0.05 vs. creatine. Number of experiments = 16.

The measurement of blood pH immediately before the death of treated animals (Figure 1) confirmed previously published data [6]. In rats treated with NH_4_Cl there was a statistically significant decrease in the pH value with respect to the control, confirming that the acidosis condition was actually induced. As in previous research [6], the acidosis condition was associated with reduced animal growth (Figure 2), including in the rats treated with creatine.

### 3.2. Antioxidant Enzyme Activities, Malondialdehyde (MDA) Production, and HSP70 Expression

**Figure 3:**
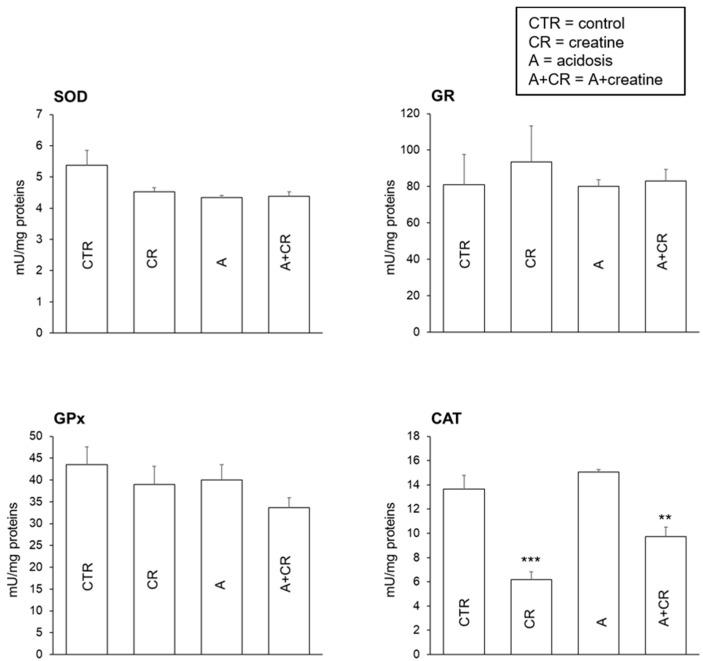
Effects of 11 d administration of 20 mM creatine on superoxide dismutase (SOD), glutathione peroxidase (GPx), glutathione reductase (GR), and catalase (CAT) activity in jejunal mucosal homogenate under control conditions and chronic acidosis. Values are means ± S.E. Number of experiments = 4 with duplicate estimation. *** *p* ≤ 0.001 vs. control and acidosis. ** *p* ≤ 0.05 vs. control and acidosis.

Figure 3 shows the activity of superoxide dismutase (SOD), glutathione peroxidase (GPx), glutathione reductase (GR), and catalase (CAT) under the different studied conditions, respectively. As shown in previous research [6], the activity of SOD, GPx, and GR did not vary significantly in chronic acidosis with respect to the control condition. Creatine did not show any effects in the control condition or in acidosis. Evidence from previous studies suggests that in murine neurons, chronic acidosis reduces the activity of GPx and GR [47], while in renal tubular cells, GPx activity is increased [48]. In rats, 6 d of oral creatine supplementation decreased the ROS content in slow- and fast-twitch skeletal muscle but did not change the expression and activity of antioxidant enzymes [49]. Our data show that none of these effects occurred in the jejunal portion of the intestine undergoing chronic acidosis, and creatine did not influence the activity of these antioxidant enzymes under basic conditions.

As previously shown [6], CAT activity (Figure 3) is not influenced by chronic acidosis. Thus, an inhibiting effect of creatine on the activity of the enzyme was evident both in the control and acidosis groups. It is known that the CAT cell level is substrate-dependent [50]. Since it has been suggested that the antioxidant effect of creatine is due to a direct scavenging action on ROS [51], it could be hypothesized that the interaction of administered creatine with ROS (H_2_O_2_) produced by normal cellular metabolism led to the observed decrease in CAT activity. The reduction of CAT activity in the presence of creatine could also be explained by taking mitochondrial metabolism into account. In fact, ROS production in the mitochondria depends strongly on the mitochondrial transmembrane potential. When mitochondrial ADP levels decrease, the membrane potential increases in association with ROS formation [52]. One of the enzymes involved in the recycling mechanism of ADP is mitochondrial creatine kinase (mt-CK), which is located in the transmembrane space of the mitochondria. This enzyme catalyzes the reaction: MgATP + Cr ↔ PCr + MgADP + H^+^. Phosphocreatine produced at the mitochondrial level is exported to the cytosol, while ADP produced at the cytosolic level is pumped into the mitochondrion. This causes an increase in ADP levels in the mitochondria and, therefore, reduces the production of ROS and H_2_O_2_. Thus, the administration of creatine could have an antioxidant role by acting through this mechanism [52].

**Figure 4:**
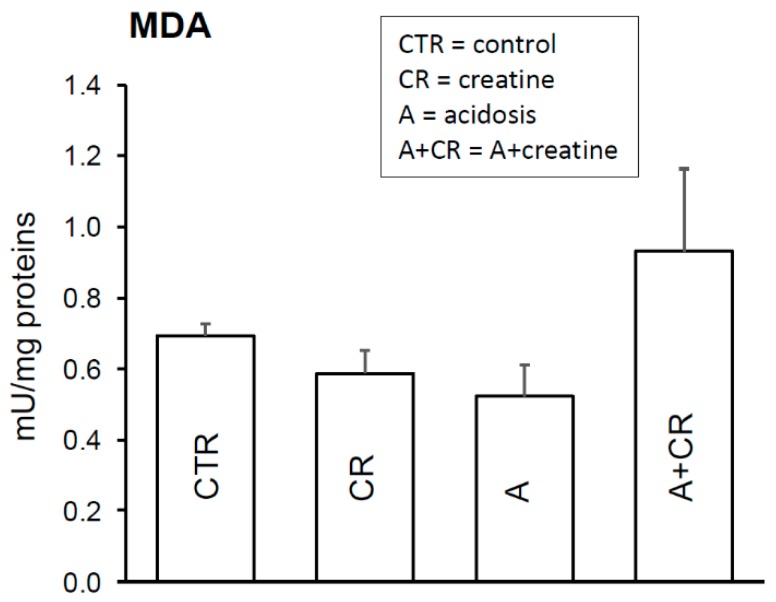
Effects of 11 d administration of 20 mM creatine on malondialdehyde (MDA) production of jejunal mucosal homogenate under control conditions and chronic acidosis. Values are means ± S.E. Number of experiments = 4 with duplicate estimation.

Lipid peroxidation is one of the detrimental consequences of oxidative damage. In fact, it elicits structural and functional damage to membranes and gives rise to several secondary products, including malondialdehyde (MDA). From Figure 4, it can be observed that the MDA levels of the jejunal mucosa did not undergo modifications in the different experimental cases, suggesting that chronic acidosis does not induce oxidative damage, and creatine does not influence the degree of lipid peroxidation in the control condition or in acidosis. Discordant data are reported in the literature on this subject. At the plasma level, for example, creatine administration is associated with a significant reduction in lipid peroxidation biomarkers [53]. Also, lipid peroxidation was shown to be reduced by creatine in the skeletal muscle of rats subjected to hyperhomocysteinemia [54]. However, the antioxidant effect of creatine found in plasma has not been observed in the liver [55], suggesting that the actions of the molecule may be different in different tissues.

**Figure 5:**
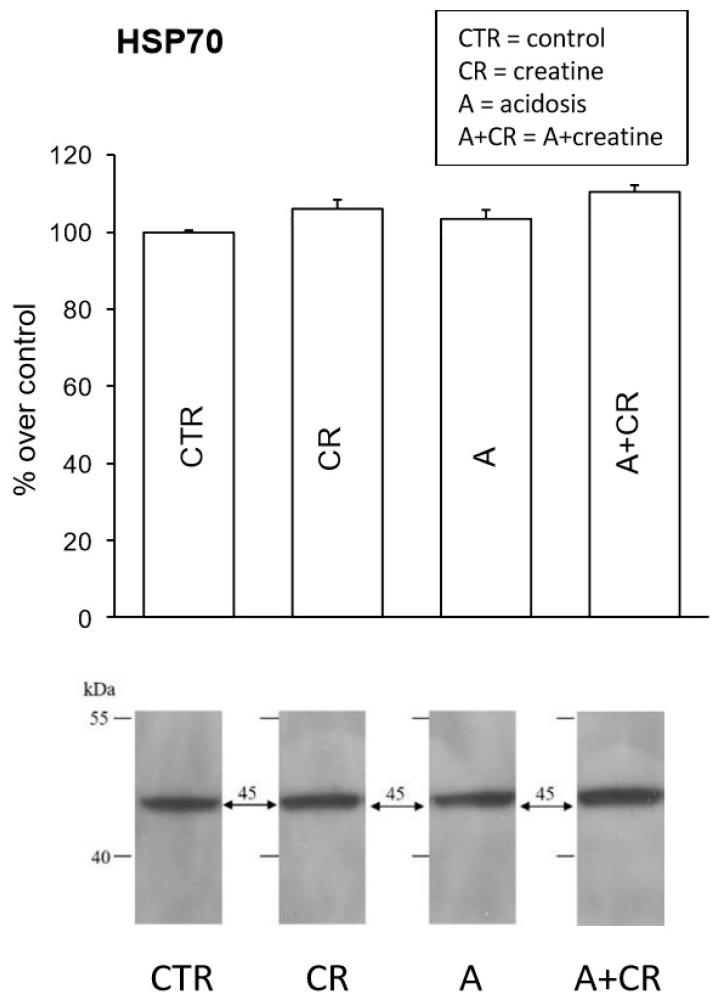
Effects of 11 d administration of 20 mM creatine on HSP70 expression in jejunal mucosal homogenates under control conditions and chronic acidosis. Western blot analysis for HSP 70 was performed on jejunum total proteins and was carried out on 4 experiments. Densitometric analysis of the bands did not reveal significant statistical differences in the intensity of the signals.

HSP70 confers stress tolerance and cytoprotection against several environmentally induced injury conditions [56,57]. Thus, the possible induction of HSP70 by creatine was investigated. HSP70 protein expression was measured using a western blot analysis (Figure 5). In all experimental conditions the presence of a band at 45 kDa, corresponding to the molecular weight of the 1A subunit of the tested protein, was observed. The related densitometric analysis showed no significant variations among all considered cases. However, we cannot exclude that there is a temporal dependence on the expression of HSP70, whose levels are notoriously modulated in a transient manner [58].

### 3.3. Expression of Na^+^/K^+^-ATPase, GLUT2, and SGLT1

To evaluate the effects of creatine treatment on intestinal function under the control and chronic acidosis conditions, we investigated the expression of some important transport proteins: Na^+^/K^+^-ATPase, GLUT2, and SGLT1.

**Figure 6:**
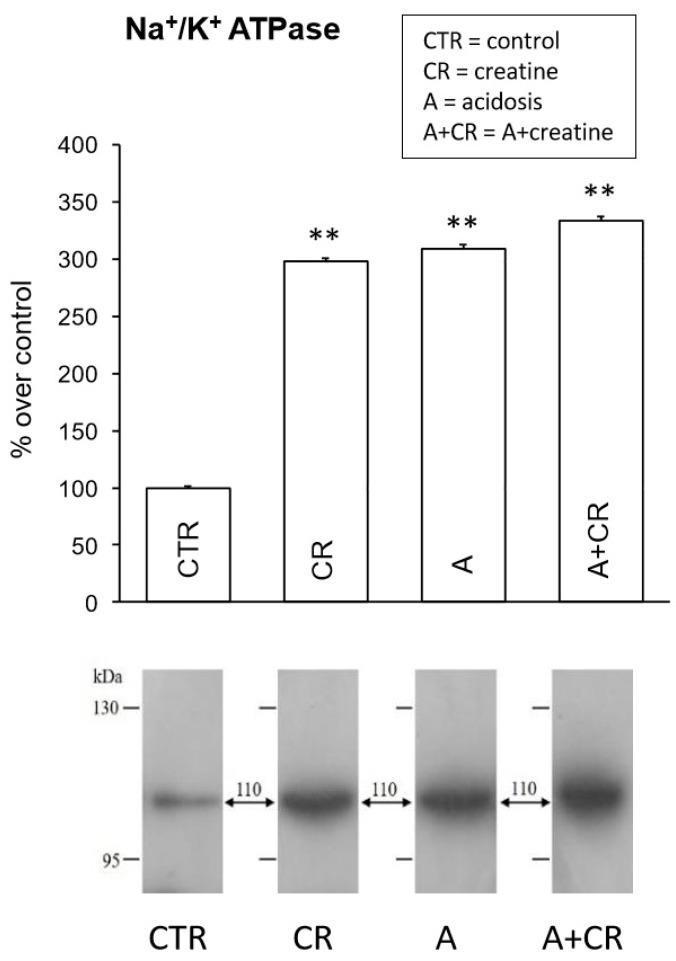
Effects of 11 d administration of 20 mM creatine on Na^+^/K^+^-ATPase expression in jejunal mucosal homogenates under control conditions and chronic acidosis. Western blot analysis for Na^+^/K^+^-ATPase was performed on jejunum total proteins and was carried out on 4 experiments. Densitometric analysis of the bands revealed a significant statistical difference in the intensity of the signals. ** *p* ≤ 0.05 vs. control.

Figure 6 shows the results of western blot assays on Na^+^/K^+^-ATPase expression. In all experimental conditions considered, the immunoblots showed the presence of a 110 kDa band, corresponding to the molecular weight of the α1 subunit of the Na^+^/K^+^-ATPase. A significant increase in the signal obtained was observed after treatment with creatine and under conditions of chronic acidosis (about +200% in both conditions), as previously observed in other tissues. In fact, it has been reported that chronic treatment with creatine induces an increase in the expression of Na^+^/K^+^-ATPase in the cerebral cortex [13], probably as a result of a functional enhancement due to the ergogenic properties of this molecule. As previously observed [6], and similarly to what was already proposed in the duodenum [59], an increase in the signal observed under conditions of chronic acidosis could be interpreted as a long-term adaptive response that is able to compensate for the reduction in Na^+^/K^+^-ATPase expression (and the consequent functional alterations of the reabsorption of ions and glucose) induced by acidosis over a short time period. Moreover, this compensatory effect would allow the acidosis itself to be counteracted, since it can be hypothesized that the increase in protein expression leads to an increase in its activity and, therefore, to the electrochemical potential gradient of Na^+^ through the plasmalemma. This could, in turn, lead to an increase in Na^+^/H^+^ exchanger activity in an attempt to resolve acid/base decompensation by bringing the pH back to the physiological value. In this regard, it should be noted that there is no additivity between the effects of chronic acidosis and creatine, so chronic treatment with creatine in the simultaneous presence of conditions of chronic acidosis would not exert any enhancement on the expression of Na^+^/K^+^-ATPase, since acidosis by itself should have induced a compensatory increase in the expression of the carrier.

**Figure 7:**
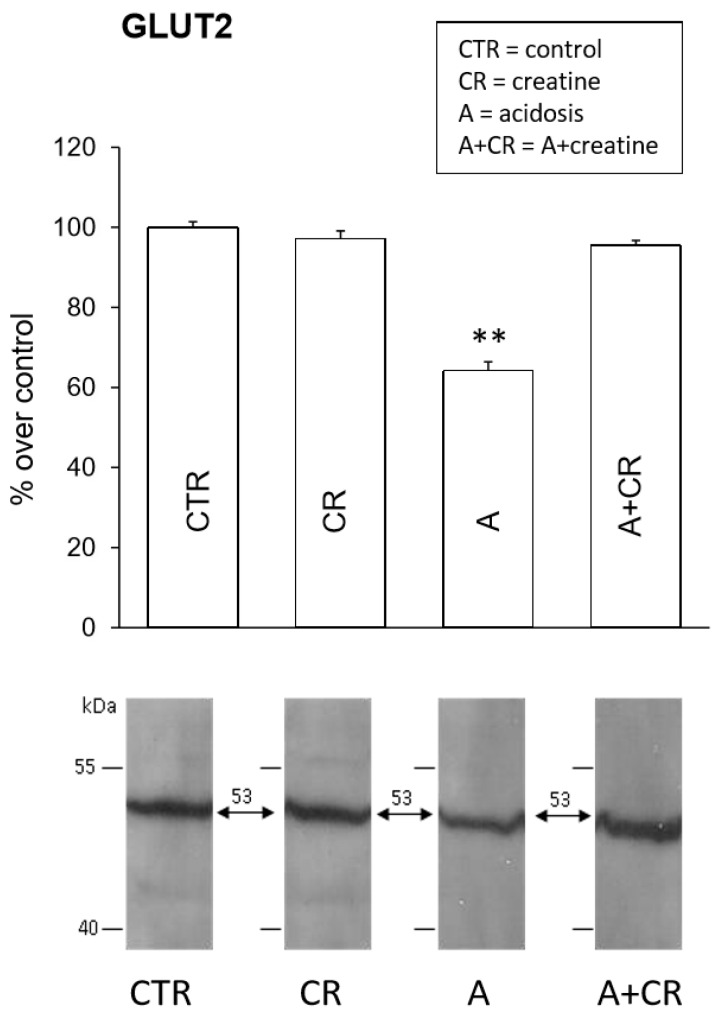
Effects of 11 d administration of 20 mM creatine on glucose transporter 2 (GLUT2) expression in jejunal mucosal homogenates under control conditions and chronic acidosis. Western blot analysis for GLUT2 was performed on jejunum total proteins and was carried out on 4 experiments. Densitometric analysis of the bands revealed a significant statistical difference in the intensity of the signals. ** *p* ≤ 0.05 vs. control.

Figure 7 shows the results of western blot assays on GLUT2 expression. In all experimental conditions, the immunoblots show the presence of a band at the expected weight of about 53 kDa. Creatine administration did not lead to any differences in the expression of GLUT2 with respect to the control condition. The molecule, therefore, does not appear to have any ergogenic effect on the levels of this carrier, at least at physiological pH. As previously shown, chronic acidosis causes a reduction in the expression of GLUT2 [6], confirming the data in the literature [60] that shows that the expression of this transporter is influenced by perturbations of the physiological conditions related to stresses of various nature. This reduction is abolished by creatine. In fact, following the simultaneous administration of NH_4_Cl and creatine, the expression levels of GLUT2 are comparable to those of the control. Under these conditions, therefore, creatine would exert a protective action on the expression of GLUT2 that is able to counteract the negative effects of chronic acidosis.

**Figure 8:**
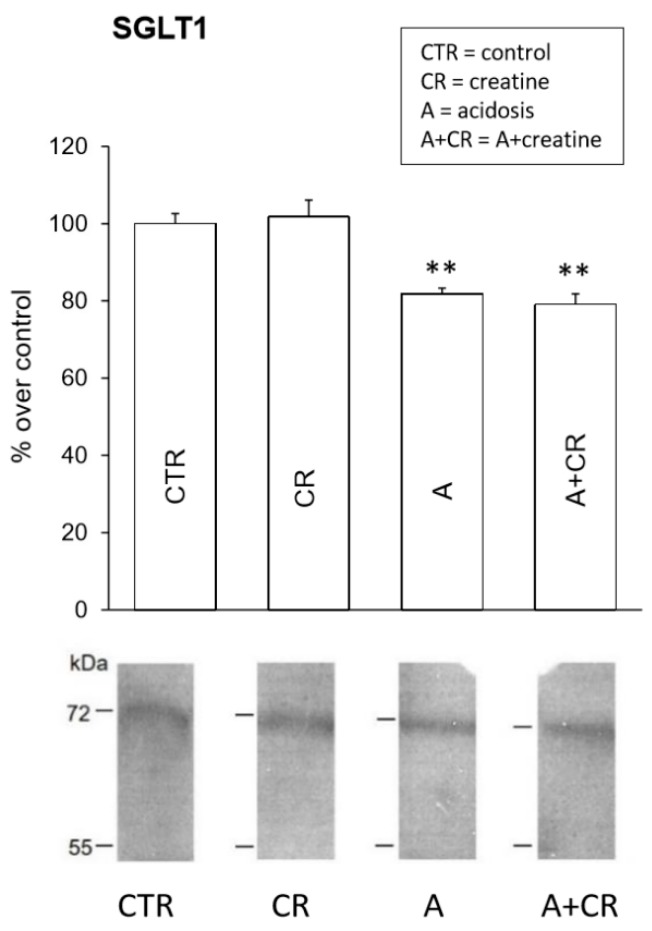
Effects of 11 d administration of 20 mM creatine on sodium-glucose transporter 1 (SGLT1) expression in jejunal mucosal homogenates under control conditions and chronic acidosis. Western blot analysis for SGLT1 was performed on jejunum total proteins and was carried out on 4 experiments. Densitometric analysis of the bands revealed a significant statistical difference in the intensity of the signals. ** *p* ≤ 0.05 vs. control.

Figure 8 shows the results of the western blot assays on SGLT1 expression. In all experimental conditions considered, the immunoblots showed the presence of a band at the expected molecular weight of 72 kDa. Following chronic treatment with creatine at physiological pH, there were no significant changes in the signal, which was instead significantly reduced (by approximately −20%) following the administration of NH_4_Cl and did not undergo further modifications following simultaneous treatment with NH_4_Cl and creatine. Chronic acidosis, therefore, led to a certain reduction in the expression of the protein, both in the absence and in the presence of creatine, which, contrary to what was observed for the Na^+^/K^+^-ATPase, did not induce any strengthening effect on the expression of SGLT1 at either physiological pH or under conditions of chronic acidosis.

## 4. Conclusions

To sum up, chronic treatment with creatine was shown to have beneficial effects on the jejunal epithelium. Creatine seems to have antioxidant properties that are realized through direct interaction of the molecule with ROS. In fact, the antioxidant status of the cell is not influenced by its administration, except for CAT, whose activity is significantly reduced both in the presence and in the absence of acidosis. The administration of creatine seems to make the tissue more resistant. In fact, its presence leads to functional strengthening of the tissue, increasing the expression of Na^+^/K^+^-ATPase. Chronic treatment with creatine also counteracts the inhibitory effect of acidosis on GLUT2, whose level of expression does not differ from the control under acidosis conditions if creatine is present. There is no involvement of HSP70 in the effects shown for creatine, as its expression does not change in its presence.

## Abbreviations

ATP	adenosine triphosphate
ANOVA	analysis of variance
CAT	catalase
Cr	creatine
EDTA	ethylenediamine tetra-acetic acid
GLUT2	glucose transporter 2
GPx	glutathione peroxidase
GR	glutathione reductase
HSP70	heat shock protein 70
NADPH	nicotinamide adenine dinucleotide phosphate
MDA	malondialdehyde
PCr	phosphocreatine
ROS	reactive oxygen species
SGLT1	Sodium-Glucose Transporter 1
SOD	superoxide dismutase
